# Supplementary material for: Single-cell RNA sequencing highlights the role of inflammatory cancer-associated fibroblasts in bladder urothelial carcinoma
Source: Nat Commun. 2020 Oct 8;11:5077. doi: 10.1038/s41467-020-18916-5 (PMC7545162; doi:10.1038/s41467-020-18916-5)
Supplement: Supplementary file 1 — Supplementary Information [file 41467_2020_18916_MOESM1_ESM.pdf]

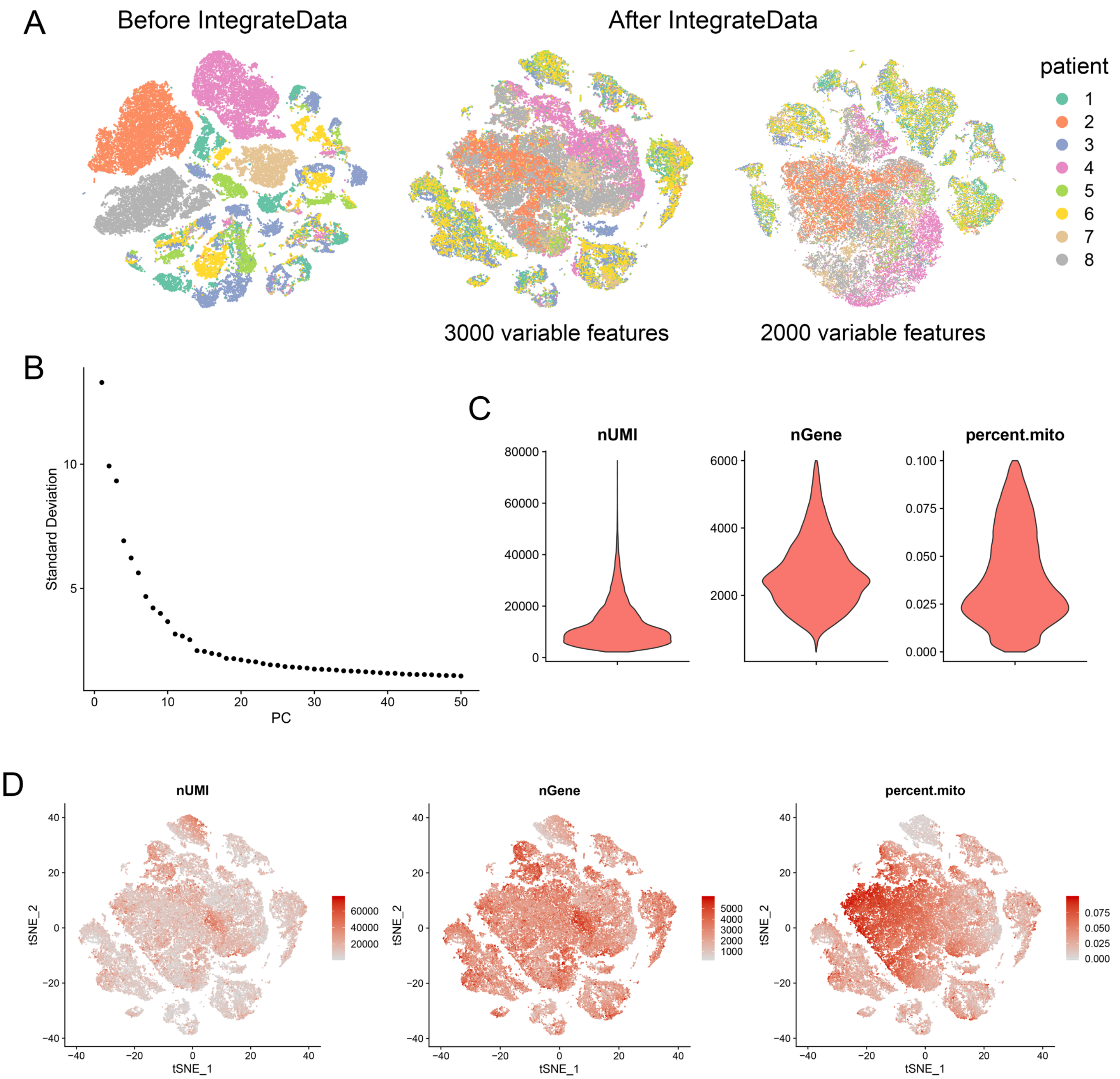

Supplementary Figure 1: Quality control of single cell sequencing data. 1A: Removing batch effect between batches. 3000 variable features (middle) and 2000 variable features (right) were used respectively. **1B:** Scree plot show top50 PCs of principle component analysis. Top30 PCs were used in downstream analysis. 1C: Vlnplot show number of UMI (nUMI), number of genes (nGene) detected and percent of mitochondrial derived transcripts (percent.mito) per single cell after quality control. **1D:** tSNE plot of single cells profiled here colored by nUMI, nGene detected, and percent.mito.

A

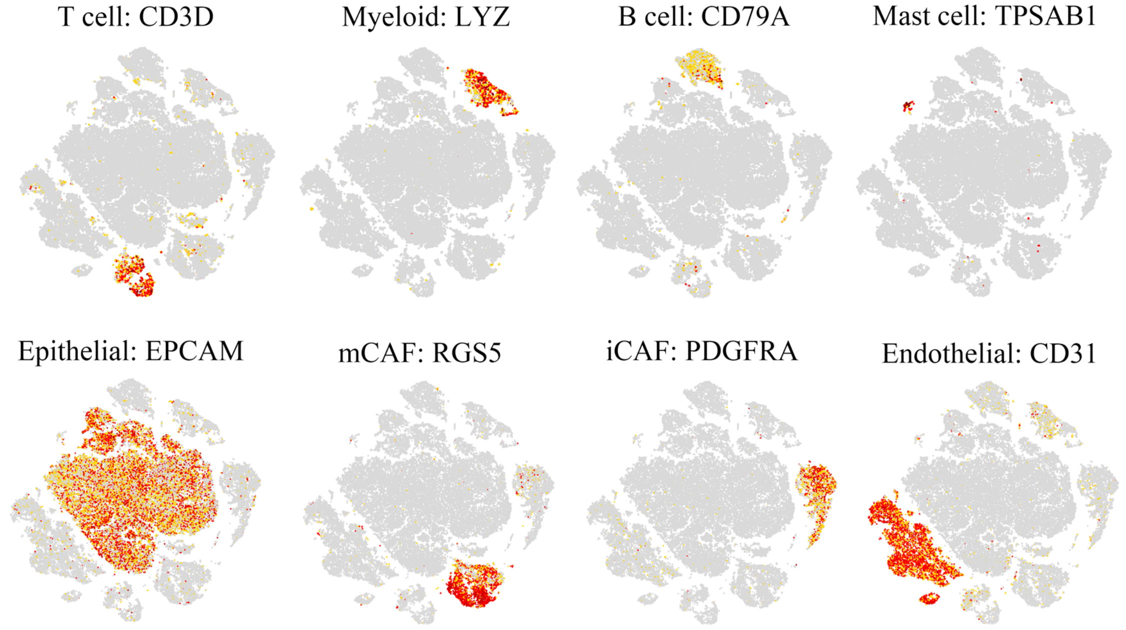

B

Sample	Number of cells	Grade
1-Normal	3356	-
3-Normal	5134	-
6-Normal	2761	-
1-Tumor	3006	Low
2-Tumor	9320	Low
3-Tumor	3463	High
4-Tumor	8226	High
5-Tumor	4218	High
6-Tumor	1818	High
7-Tumor	3854	High
8-Tumor	7565	High
**Total**	**52721**	

C

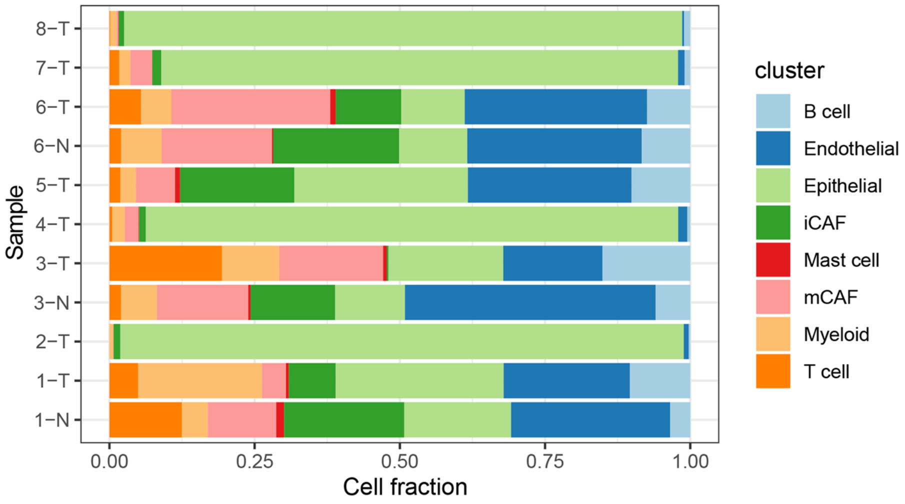

D

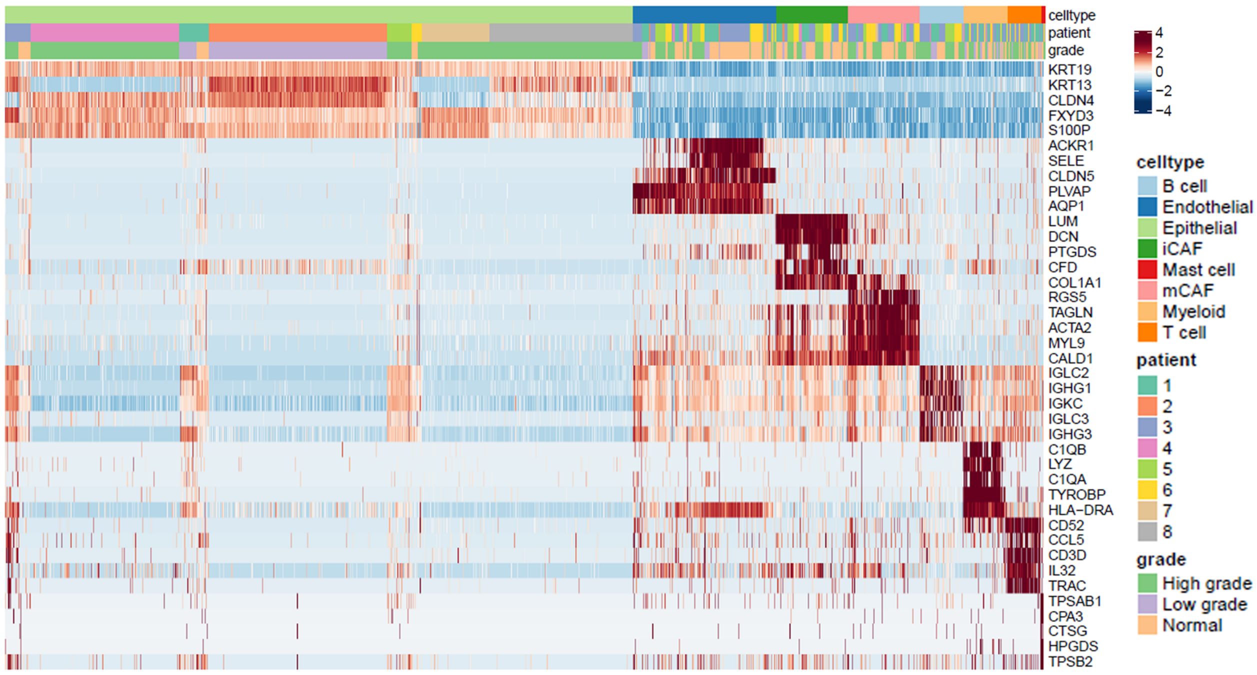

Supplementary Figure 2: Identification of major cell types in BC TME. 2A: Expression of marker genes for major cell types. Eight major cell types were identified: epithelial (EPCAM+) cells; endothelial (CD31+) cells; two types of fibroblasts (COL1A1+) – inflammatory cancer-associated fibroblasts (iCAFs) (PDGFRA+) and myo-CAFs (mCAFs) (RGS5+); B cells (CD79A+); myeloid cells (LYZ+); T cells (CD3D+); and mast cells (TPSAB1+). **2B:** Clinical information and number of cells profiled of every sample involved in the presenting work. **2C:** The fraction of major cell types originated from the 3 non-malignant samples and 8 tumor samples. **2D:** Heatmap of top 5 marker genes of every major cell types. Shown are row z-score.

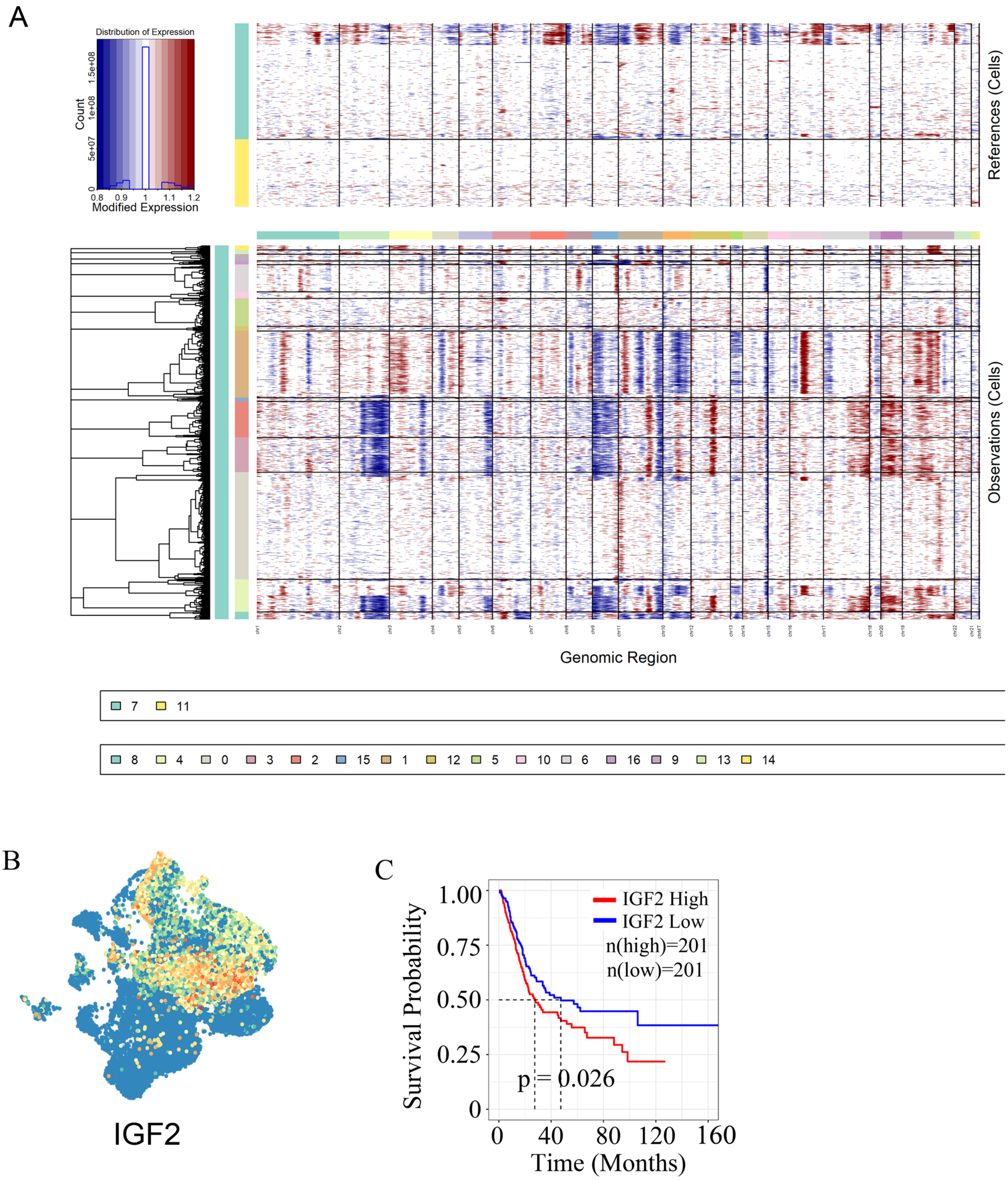

Supplementary Figure 3A: Copy number variations (CNVs) evaluated per cell by InferCNV. Two normal-derived epithelial clusters were used as control group. **3B:** Expression of IGF2 for epithelial cells. **3C:** Kaplan-Meier survival curve for IGF2 in TCGA BLCA cohort, expression of IGF2 was corrected by EPCAM expression level with GEPIA2 method. P value: log-rank p value.

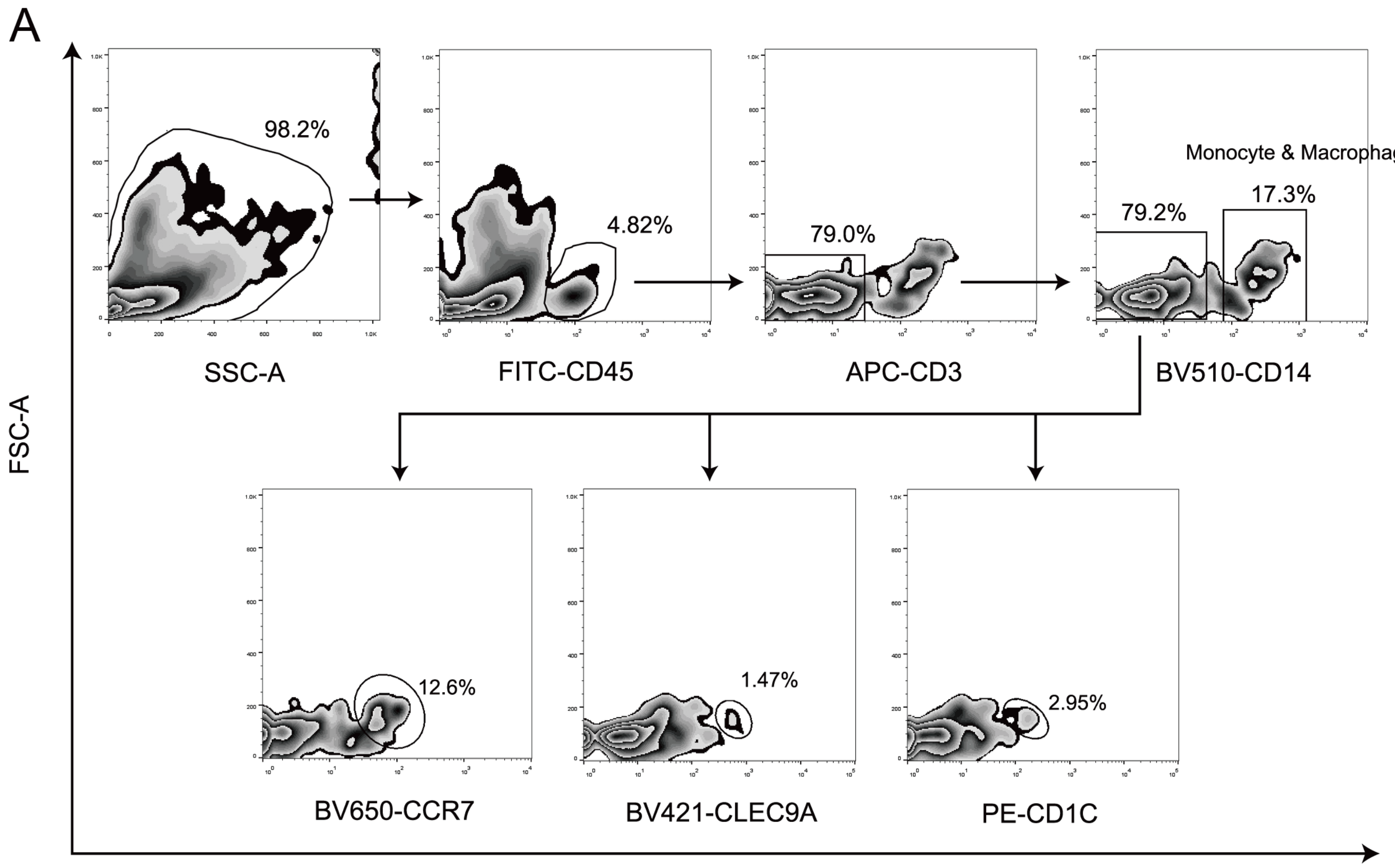

Supplementary Figure 4A: Flow cytometry confirmed the Monocyte and DC subgroups in bladder carcinoma microenvironment.

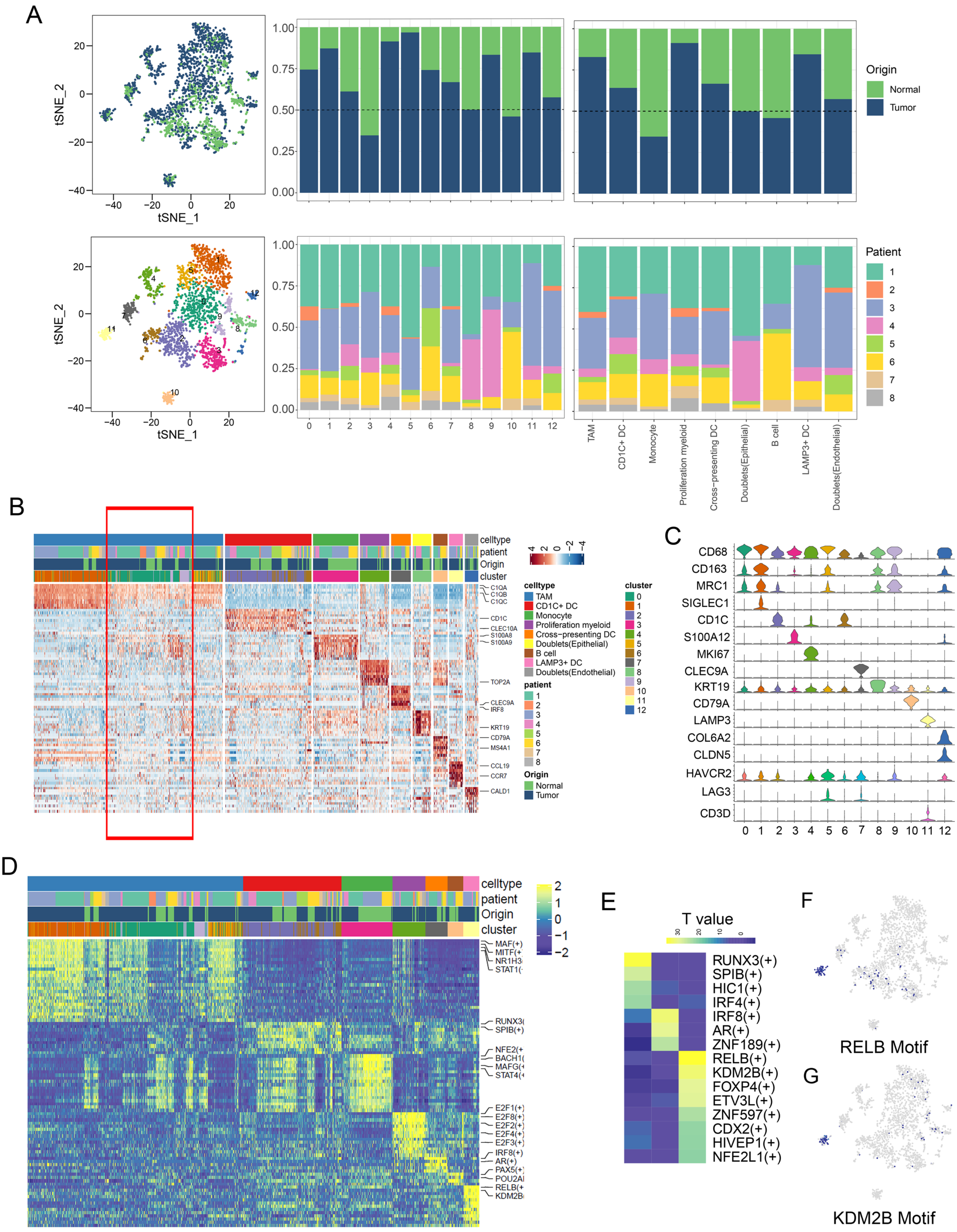
A
tSNE_1
tSNE_2
Origin
Normal
Tumor
Patient
TAM
CD1C+ DC
Monocyte
Proliferation myeloid
Cross−presenting DC
Doublets(Epithelial)
B cell
LAMP3+ DC
Doublets(Endothelial)
B
celltype
patient
Origin
cluster
C1QA
C1QB
C1QC
CD1C
CLEC10A
S100A8
S100A9
TOP2A
CLEC9A
IRF8
KRT19
CD79A
MS4A1
CCL19
CCR7
CALD1
C
CD68
CD163
MRC1
SIGLEC1
CD1C
S100A12
MKI67
CLEC9A
KRT19
CD79A
LAMP3
COL6A2
CLDN5
HAVCR2
LAG3
CD3D
D
E
T value
RUNX3(+)
SPIB(+)
HIC1(+)
IRF4(+)
IRF8(+)
AR(+)
ZNF189(+)
RELB(+)
KDM2B(+)
FOXP4(+)
ETV3L(+)
ZNF597(+)
CDX2(+)
HIVEP1(+)
NFE2L1(+)
F
RELB Motif
G
KDM2B Motif

Supplementary Figure 5A: tSNE plot of LYZ+ cells colored by origin (left, up) and clusters (left, down). For cell clusters or cell subgroups, fractions of cells originating from tumor or non-malignant tissues and different patients were also offered in the right. **5B:** Heatmap of top 5 marker genes of every cell subgroups. Shown are row z-score. **5C:** Violin plots of selected cell type-specific genes. **5D:** Activation of TF motifs evaluated by SCENIC could also identify cell subgroups. Shown are row z-score. **5E:** Top differentially activated TF motifs between DC subgroups. Shown are t-values from a linear model. 5**F-5G:** AUC of top 2 LAMP3+ DC specific TF motifs.

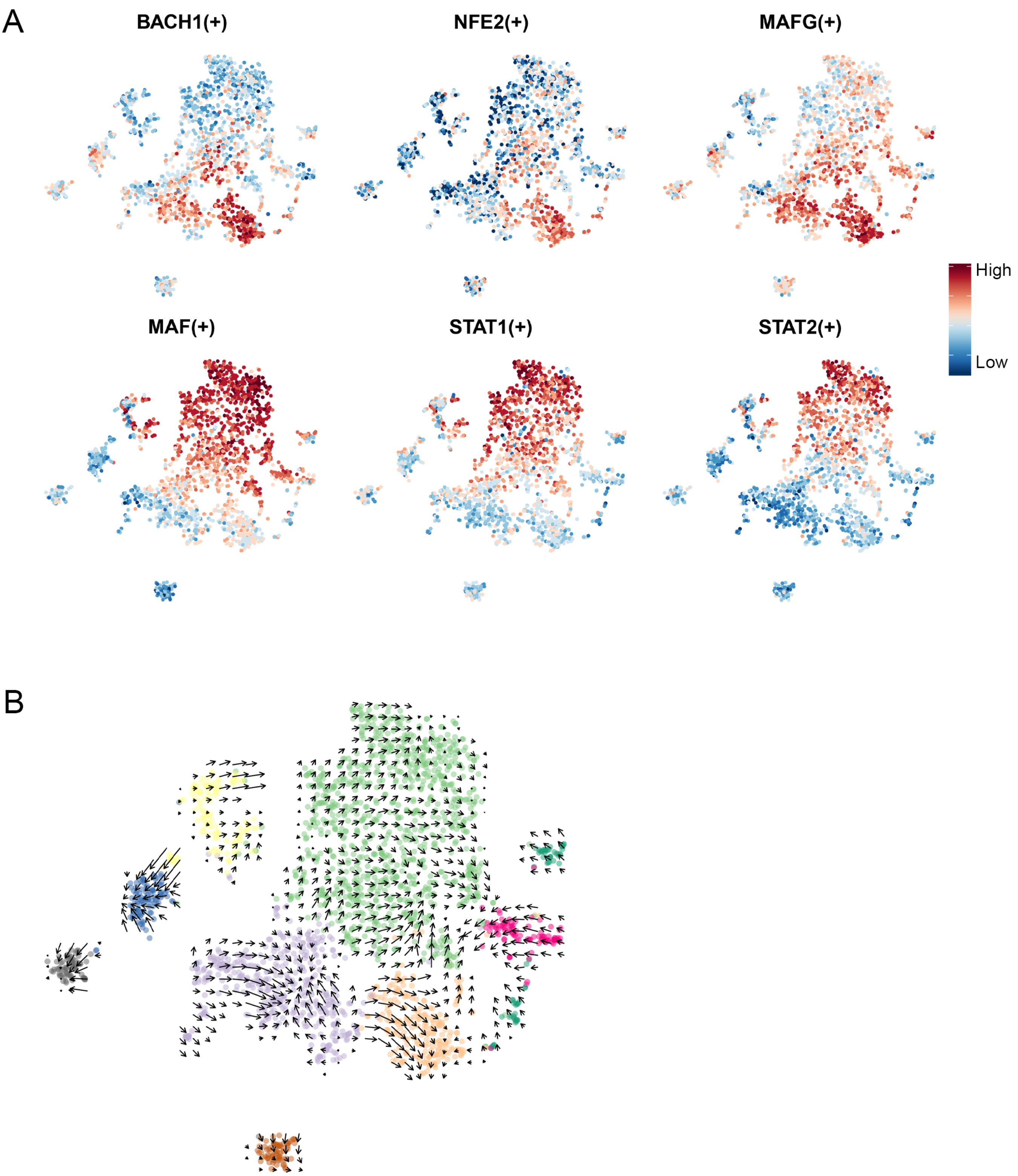

Supplementary Figure 6A: AUC of potential regulons take part in polarization of monocyte. 6B: RNA velocity analysis of Myeloid lineage.

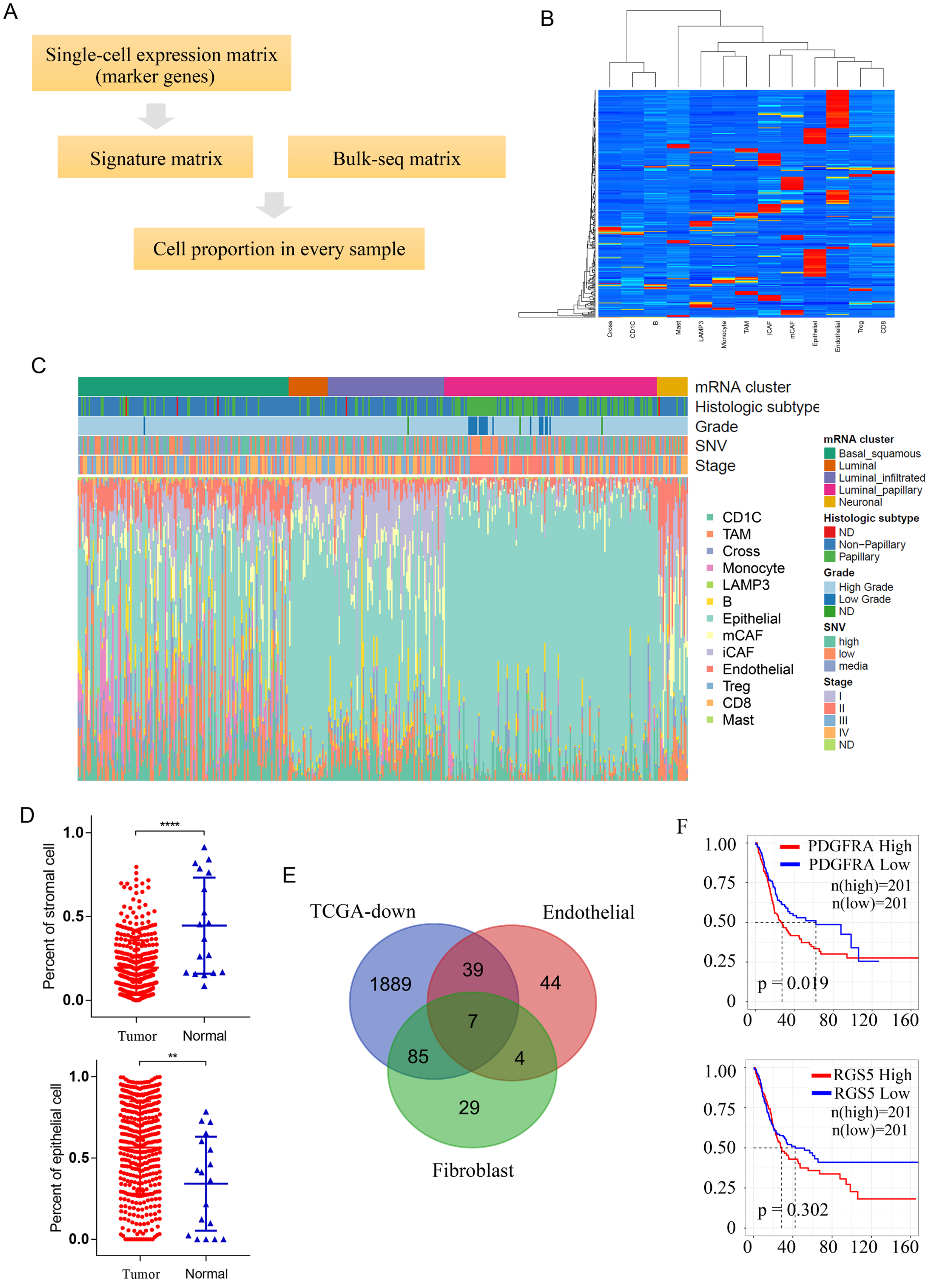
A
Single-cell expression matrix (marker genes)
Signature matrix
Bulk-seq matrix
Cell proportion in every sample
B
Cross
CD1C
B
Mast
LAMP3
Monocyte
TAM
iCAF
mCAF
Epithelial
Endothelial
Treg
CD8
C
mRNA cluster
Histologic subtype
Grade
SNV
Stage
CD1C
TAM
Cross
Monocyte
LAMP3
B
Epithelial
mCAF
iCAF
Endothelial
Treg
CD8
Mast
mRNA cluster
Basal_squamous
Luminal
Luminal_infiltrated
Luminal_papillary
Neuronal
Histologic subtype
ND
Non−Papillary
Papillary
Grade
High Grade
Low Grade
ND
SNV
high
low
media
Stage
I
II
III
IV
ND
D
Percent of stromal cell
****
Tumor
Normal
Percent of epithelial cell
**
Tumor
Normal
E
TCGA-down
Endothelial
Fibroblast
1889
39
44
7
85
4
29
F
PDGFRA High
PDGFRA Low
n(high)=201
n(low)=201
p = 0.019
RGS5 High
RGS5 Low
n(high)=201
n(low)=201
p = 0.302

Supplementary Figure 7A: Workflow of the estimation of relative cell abundance with CIBERSORTx. **7B:** Signature matrix created by CIBERSORTx. 7**C:** Fraction of tumor infiltrated cells was associated with molecular subtypes. 7**D:** Tumor sample contains less stromal cells and more epithelial cells in TCGA BLCA cohort. Two side student's t-test was conducted. **: $p < 0.01$, ****: $p < 0.0001$. 7**E:** Venn plot shown overlapped DEGs between TCGA down-regulated genes, endothelial markers and Fibroblast markers. 7**F:** Kaplan-Meier survival curve of iCAFs and mCAFs specific genes. P: log-rank p value.

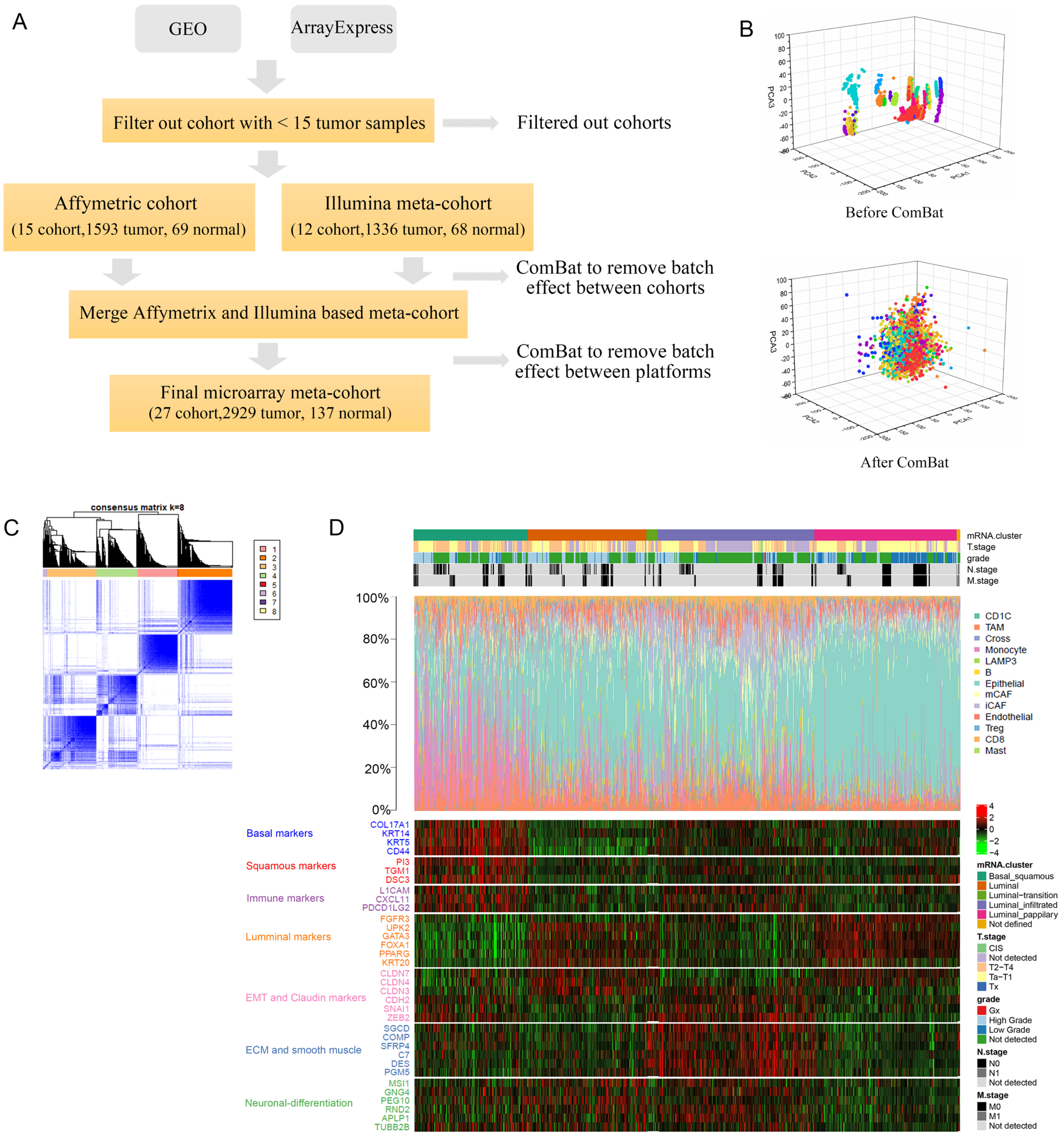

Supplementary Figure 8A-8B: Workflow of elimination of batch effect between different BC cohort. 8**C:** Clustering of tumor samples with ConsensusClusterPlus identified 5 different molecular subtypes. 8**D:** Fraction of cells in every sample (up) and expression of known marker genes of molecular subtypes.

A

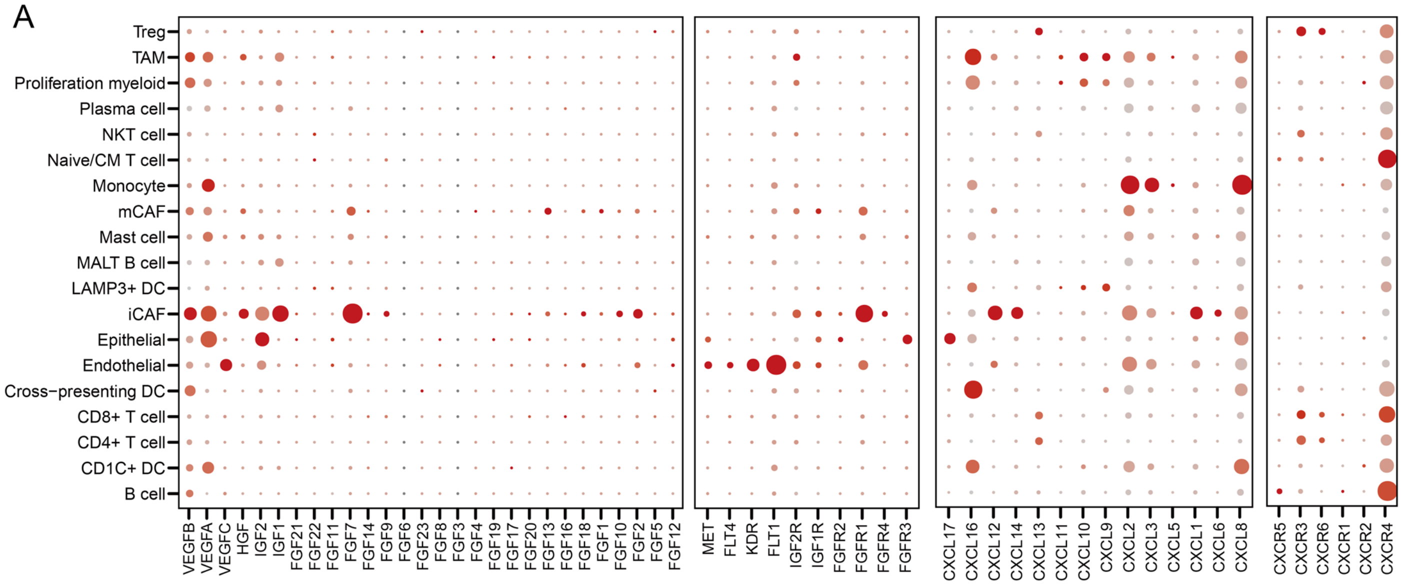

Supplementary Figure 9: Expression of growth factors, chemokines and their receptors across cell subgroups.

Supplementary Table 1: Clinical information of bladder carcinoma and non-malignant tissues

SampleID	PatientID	Cell Num before QC	Cell Num after QC	gender	age	Grade	Invasiveness	Type of surgery	Tumor size	Therapy
L-T1	1	3267	3006	M	67 ys	low	Noninvasive	TURBT	1.9 cm	Primary tumor without pretreatment
L-T2	2	10049	9320	M	70 ys	low	Noninvasive	TURBT	2.5 cm	primary tumor with no pretreatment
H-T1	3	3584	3463	M	63 ys	high	Noninvasive	Cystectomy	3.5 cm	primary tumor with no pretreatment
H-T2	4	8342	8226	F	59 ys	high	Noninvasive	Cystectomy	4.7 cm	primary tumor with no pretreatment
H-I-T1	5	4467	4218	M	57 ys	high	Invasive	Cystectomy	5.1 cm	primary tumor with no pretreatment
H-I-T2	6	3180	1818	M	75 ys	high	Invasive	Cystectomy	4.3 cm	primary tumor with no pretreatment
H-I-T3	7	4270	3854	M	77 ys	high	Invasive	Cystectomy	4.5 cm	primary tumor with no pretreatment
H-I-T4	8	8123	7565	F	72 ys	high	Invasive	Cystectomy	4.1 cm	primary tumor with no pretreatment
N1	1	3459	3356	M	67 ys	-	-	TURBT	-	-
N2	6	3334	2761	M	75 ys	-	-	Cystectomy	-	-
N3	3	5310	5134	M	63 ys	-	-	Cystectomy	-	-

Supplementary Table 2: Top Cell type Specific Motifs of Myeloid Lineage

Celltype	TOP specific TF
TAM	MAF(+)
TAM	MITF(+)
TAM	NR1H3(+)
TAM	STAT1(+)
TAM	USF2(+)
TAM	STAT2(+)
TAM	CEBPA(+)
TAM	ETV5(+)
TAM	TFEC(+)
TAM	MAFB(+)
TAM	CREB3L2(+)
TAM	ARNT(+)
CD1C+ DC	RUNX3(+)
CD1C+ DC	SPIB(+)
CD1C+ DC	HIC1(+)
CD1C+ DC	IRF4(+)
Cross-presenting DC	IRF8(+)
Cross-presenting DC	AR(+)
Cross-presenting DC	ZNF189(+)
Monocyte	NFE2(+)
Monocyte	BACH1(+)
Monocyte	CREB5(+)
Monocyte	MAFG(+)
Monocyte	STAT4(+)
Monocyte	NFKB2(+)
LAMP3+ DC	RELB(+)
LAMP3+ DC	KDM2B(+)
LAMP3+ DC	FOXP4(+)
LAMP3+ DC	ETV3L(+)
LAMP3+ DC	ZNF597(+)
LAMP3+ DC	CDX2(+)
LAMP3+ DC	HIVEP1(+)
LAMP3+ DC	NFE2L1(+)
